# Quantity versus Quality: Chlorhexidine Bathing Adequacy Assessments in 3 High-Risk Units

**DOI:** 10.1017/ash.2024.138

**Published:** 2024-09-16

**Authors:** Michelle Doll, Barry Rittmann, Patrick Ching, Kaila Cooper, Yvette Major, Gonzalo Bearman

**Affiliations:** Virginia Commonwealth University; Nursing VCU Health; VCUHS; Editor in Chief ASHE

## Abstract

**Background:** Chlorhexidine gluconate bathing (CHGB) prevents healthcare associated infections (HAIs). CHGB quality is rarely assessed; prior studies identified that concentrations of CHG can be suboptimal, particularly at the neck, and if rinsed after application. In the setting of increased HAI rates on 3 high-risk units, we evaluated CHG skin concentrations, comparing results to bathing documentation and patient reports as part of a quality improvement initiative. **Methods:** All patients admitted to 3 high-risk units were swabbed for CHG concentration testing at the neck, bilateral upper arms, and groin. Swabs were processed using a semi-quantitative colorimetric CHG assay. A threshold of 0.001875% CHG was used to determine adequacy based on prior studies. Adequacy was assessed by body site, timing of bath, and patient-reported skin care activities using Chi-square tests in SAS 9.4. Per hospital policy, all admitted patients are bathed daily with 2% CHG pre-packed wipes. Patients without a documented CHGB for the duration of the admission were excluded. **Results:** CHG testing was completed on 63 patients: 23 on medical ICU, 18 surgical ICU, 22 oncology ward, yielding 249 samples. Only ward patients could report the time of last CHGB, which agreed with nursing documentation for 12/21(57%) Adequacy by sample was no different across units: 59/88(67%) Oncology, 68/90(76%) MICU, 56/71(79%) SICU, p=0.2091. Site adequacy was different by site: neck 36/63(57%), left arm 49/62(79%), right arm 50/62(81%), groin 48/62(77%), p=0.0083. Samples taken from the 11 patients with > = 24 hours since last CHGB were more likely to be below threshold concentration: 19/47(40%) versus 47/202(23%) not adequate in the recent treatment grouping. Three patients reported showering soon after the CHGB and 8 patients used moisturizing lotion. The percent of samples below threshold for the showering patients (6/12, 50%) and lotion-users (11/32, 34%) were not significantly different from the non-showering or non-lotion using patient samples (p=0.0588 and 0.2800 respectively). **Conclusion:** In a facility with longstanding daily CHGB policies in place, 66/249 samples from 63 patients lacked adequate concentrations of CHG for optimal HAI prevention. Even in patients with recent CHGB, 23% of sites tested revealed inadequate levels of CHG, while 60% of those overdue for CHGB kept adequate concentrations. Reliable implementation strategies are required for CHGB so as to ensure maximal infection prevention impact.

